# Effect of sea tangle extract on the quality characteristics of reduced-salt, low-fat sausages using pre-rigor muscle during refrigerated storage

**DOI:** 10.5713/ab.23.0150

**Published:** 2023-08-23

**Authors:** Geon Ho Kim, Koo Bok Chin

**Affiliations:** 1 Department of Animal Science, Chonnam National University, Gwangju 61186, Korea

**Keywords:** Functional Properties, Pork Sausage, Reduced-salt, Sea Tangle Extract, Shelf-life

## Abstract

**Objective:**

The aim of this study was to investigate quality characteristics of reduced-salt, low-fat pork sausage (PS) using pre-rigor muscle and sea tangle extract (STE) to reduce salt level of sausages during refrigerated storage.

**Methods:**

Pork ham was prepared with pre-rigor and post-rigor muscle from the local market. Sausages using post-rigor muscle were manufactured with the 1.5% of salt content, and samples with pre-rigor muscle were processed by different salt concentrations (0.8%). Accordingly, PSs were prepared in 4 treatments (REF, PS with 1.5% of salt using post-rigor muscle; CTL, PS with 0.8% of salt using pre-rigor muscle; TRT1, PS with 0.8% of salt and 5% of STE using pre-rigor muscle; TRT2, PS with 0.8% of salt and 10% of STE using pre-rigor muscle). For the evaluation of quality characteristics and shelf-life of reduced-salt PS, pH and color values, cooking loss (%), expressible moisture (%), textural properties, lipid oxidation (thiobarbituric reactive substances), protein denaturation (volatile basic nitrogen), and microbiological analysis (total plate counts and *Enterobacteriaceae* counts) were determined.

**Results:**

The pH and temperature of pre-rigor raw pork ham were higher than those of post-rigor pork ham. Hardness of TRT2 was higher than that of REF or CTL. TRT2 had higher gumminess and chewiness than CTL. TRT1 and TRT2 had lower volatile basic nitrogen than CTL. Total plate counts of TRT2 were lower than those of CTL. Expressible moisture values of TRT1 and TRT2 were similar to those of REF. The addition of STE into PS improved functional properties and shelf-life of PS.

**Conclusion:**

Reduced-salt PS containing pre-rigor muscle and STE had similar functional properties to those of regular-salt ones, while containing approximately 47% less salt compared to regular-salt level.

## INTRODUCTION

The addition of salt in the manufacture of meat products is essential to have good quality characteristics since salt can improve flavor and taste of meat products and inhibit growth of microorganisms during storage [1]. During sausage manufacture, myofibrillar proteins (salt-soluble proteins) can be extracted by adding salt and cutting the meat, thus producing desirable texture during cooking. This extraction procedure can improve water-holding capacity, cooking yield, and texture properties which are important factors that determine the quality of meat products.

Although salt has such excellent functions for processed meats, its addition level should be reduced because excessive salt intake can increase the risk of hypertension, which is a major factor of coronary artery and cardiovascular diseases [2]. Thus, it is desirable to reduce the intake of salt to prevent these diseases. The European Food Safety Authority (EFSA) recommends a salt intake of 5 g per day for adults [3]. World Health Organization [4] reported that adequate sodium intake should be less than 2,000 mg/d. However, average salt intake for the most people was 9–12 g per day [5]. Thus, strategies to reduce the salt addition in meat products are highly recommended.

Hot boning is a deboning technique to fabricate muscle from carcass without a chill processing before rigor-mortis. Pre-rigor muscle produced by hot boning show better functional characteristics than post-rigor ones. Pre-rigor muscle has higher pH and temperature than post-rigor ones due to incomplete metabolism of hot carcass. Thus, processed meats using pre-rigor muscle could have superior processing properties. Verma and Banerjee [6] stated that pre-rigor meat had great functional properties such as binding ability, water-holding capacity, and extractability of myofibrillar proteins in their review paper. Pork hams using pre-rigor muscle had higher cooking yield and water-holding capacity than those using post-rigor ones [7]. Puolanne and Terrell [8] reported that reduced-salt frankfurter-type sausages with pre-rigor pork had no negative effects on physico-chemical and sensory properties. Thus, the use of pre-rigor muscle would be useful for developing reduced-salt meat products.

Sea tangle (*Saccharina japonica*) is brown algae that grows on the coast of East Asia. It has a flavor compound that is used as a seasoning to improve sensory properties of several foods. Glutamic acid and aspartic acid could impart umami flavor to foods. They are the most abundant amino acids in sea tangle [9]. In addition, sea tangle contains alginic acid, which can inhibit microbial growth of *Staphylococcus* and *Escherichia coli* [10]. Due to these characteristics, sea tangle, might be suitable for use in reduced-salt meat products to improve sensory properties and extend shelf-life of these products. Several studies have reported that the application of seaweeds including sea tangle to meat products can improve functionalities and shelf-life of these meat products, including mixed meat (beef, pork, and chicken) patties [11], breakfast sausages [12], reduced-fat pork patties [13], reduced-salt frankfurters [14], and emulsion-type sausages [15]. However, no study has reported pre-rigor muscle added with sea tangle extract (STE). Therefore, the objective of this study was to improve quality characteristics of reduced-salt pork sausages using by pre-rigor muscle added with STE for developing reduced-salt meat products.

## MATERIALS AND METHODS

### Materials

Raw pork ham (Castrated, 1st Korean grade, Landrace× Yorkshire×Duroc) was purchased from a local wholesale store (Hyundai distribution, Gwangju, Korea). Pre-rigor pork ham muscle was taken from carcasses within 1 h after slaughter without chilling (pH 6.32±0.37, 10.3°C±4.25°C of internal temperature). Post-rigor muscle was obtained from chilled carcasses at more than 24 h after slaughter (pH 5.62±0.17, 31.4°C±5.72°C of internal temperature). A whole pork ham muscle of each type (pre- and post-rigor) fabricated from different half carcasses was used per a sausages manufacture. Total 6 whole pork ham muscles from half carcasses (2 rigor states×3 replication) were allocated for sausages preparation in this study. Raw pork ham with external fat and connective tissue trimmed off was chopped with a meat grinder (M-12S; Korea Fuji Kogyo, Busan, Korea) and used to manufacture sausages. Dried sea tangle (Wando, Korea) was purchased from a local supermarket (Gwangju, Korea) and used to prepare water extract. Water and dried sea tangle were mixed at a ratio of 9:1 and heated at 90°C for 2 h. STE was filtered through a sieve (Testing sieve AST 50 300 μm; ChungGye Industrial Mfg., Co, Seoul, Korea). Filtered STE (1.75%±0.02% of salinity) was then used to manufacture pork sausages. A 5% and 10% addition might be increased the salt level by 0.0875% and 0.175% along with the original salt level and this might not affect the sausage characteristics.

### Experimental design

The formulation of manufactured pork sausage is shown in Table 1. Manufacture of sausages was triplicated at different days as a replication. Total 192 samples (2 samples per treatment×8 experimental treatment groups×4 samples per storage time×3 replication) were prepared for present study using a randomized design. Treatment groups were designated with different additional levels of salt, rigor states of raw pork ham, STE, and packaging types. Positive control treatment (REF) using post-rigor muscle was prepared with 1.5% salt added, whereas negative control (CTL) used a combination of 0.8% salt content and pre-rigor muscle without STE. TRT1 and TRT2 were sea tangle treated groups with 0.6% salt added using pre-rigor raw meat since approximately 0.2% of salt was from cure blend at the target salt level of 0.8%. They were prepared using STE instead of ice water of 5% (TRT1) or 10% (TRT2). Each formulation (n = 4) was packaged by applying different packaging types (n = 2), vacuum packaging (VAP) or modified atmosphere packaging (MAP), and the total number of treatment groups was eight (n = 8).

### Preparation of pork sausages

First, raw meat batter for pork sausage was prepared by comminuting ground raw pork ham with ice water, fat replacer, and STE (TRT1 and TRT2). It was mixed with curing agents such as salt, sodium tripolyphosphate, sodium nitrite, and sodium erythorbate. The meat batter and remaining water were then mixed to complete the manufacture of raw meat batter. After sausage batter was mixed completely, it was stuffed with polyvinylidene chloride and cooked in a water bath (WB-22; Daihan Scientific, Seoul, Korea) until the internal temperature reached 72°C. After cooling the cooked sausage, either VAP into polyethylene/nylon bag or MAP (30% CO_2_: 70% N_2_) into polyethylene terephthalate tray (wrapped by heat-sealing on a barrier polyester film) was performed to determine if there were any differences between packaging types during storage at 10°C for up to 8 wks. Four treatment groups (REF, CTL, TRT1, and TRT2) were cooked simultaneously without separate batches, and two samples per treatments were randomly allocated and packaged together in two packaging types (VAP and MAP). During storage, physicochemical and textural properties of sausages were measured at 0, 2, 4, and 8 wks of storage.

### pH values

The pH values of pork sausages as affected by salt level, addition level of sea tangle and rigor state were determined with a pH meter (Model 340; Mettler-Toledo, Greifeense, Switzerland). Calibration of pH determination was performed through standard curve calculated from pH 4.01 and pH 7.00 buffer. Determination of pH was carried out five times for each treatment. The mean of these five pH measurements was calculated and reported.

### Color (CIE L*, a*, b*) values

Color values of pork sausage were measured with a Commission Internationale de l’Eclairage (CIE) color reader (CR-10; Minolta, Tokyo, Japan) with illuminant D65, 10° standard observer and 8 mm measuring aperture. Measurements of lightness (CIE L*), redness (CIE a*), and yellowness (CIE b*) were performed six times for each sample. For maintenance of determination, standardization was carried out by checking color values of a white flat plate (CIE L* = 94.8, CIE a* = 1.0, CIE b* = 0.1).

### Cooking loss

Stuffed sausage batter weight was measured before cooking. After cooking, cooked sample was cooled in an ice. The moisture in the casing was removed and sausages were weighed again. Cooking loss percentages (CL, %) were calculated with the following formula:


$$\text{Cooking loss }(\text{CL},\%)=100-\left(\frac{Sample\;weight\;after\;heating\;(g)}{Sample\;weight\;before\;heating\;(g)}\times 100\right)$$

### Texture profile analysis

Sausage samples were cut with a diameter of 1.25 cm and height of 1.3 cm for measuring texture profile analysis (TPA). Instron Universal Testing Machine (Model 3344; Canton, MA, USA) was used with a with a compression probe and a load cell of 50 kg at a speed of 300 mm/min for determining hardness (gf), springiness (mm), gumminess, and chewiness of each treatment group 10 times.

### Expressible moisture

Sausage samples for expressible moisture (EM, %) were used in the shape of rectangular parallelepiped (1.5 g). They were wrapped with filter papers (Whatman #3; GE Healthcare, Little Chalfont, UK) and placed in conical tubes (SPL Life Science, Pocheon, Korea). Samples were centrifuged at 1,660×g for 15 min using a table top centrifuge machine. EM values were calculated as a percentage by substituting samples and expressed water weight of filter paper derived the following formula:


$$\text{Expressible moisture }(\text{EM},\%)=\frac{Expressed\;water\;weight\;of\;filter\;paper\;(g)}{Sample\;weight\;(g)}\times 100$$

### Purge loss

Packaged and unpackaged sample weight were measured during refrigerated storage. Determination of unpacked sample weight was performed after removing exuded moisture from the surface of sausages and package. Purge loss (PL, %) values were derived using the formula shown below:


$$\text{Purge loss }(\text{PL},\%)=\frac{Packaged\;sample\;weight-unpackaged\;sample\;weight}{Packaged\;sample\;weight}\times 100$$

### Thiobarbituric reactive substances

Thiobarbituric reactive substances (TBARS, mg MDA/kg) values of sausages were measured according to method of Sinnhuber and Yu [16]. Briefly, a mixture of ground sausage sample (2 g), thiobarbituric acid solution (5 mM, 3 mL), and trichloroacetic acid (17 mL) was heated in a water bath at 100°C for 30 min. After samples cooled down, the supernatant (5 mL) and chloroform (5 mL) were blended for 1 min and centrifuged at 200×g for 5 min. Then the supernatant of each sample (3 mL) and petroleum ether (1 mL) were mixed for 1 min and centrifuged at 200×g for 10 min. Finally, the optical density of the precipitation was determined with a spectrophotometer (Model UV-1601; Shimadzu, Kyoto, Japan) at a wavelength of 532 nm. TBARS values were calculated by the formula below:


$$\text{TBARS value }(\text{mg MDA}/\text{kg of sample})=\frac{Optical\;density\;value\times 9.48}{Sample\;weight\;(g)}$$

### Volatile basic nitrogen

Volatile basic nitrogen (VBN) values of sausage samples were determined according to the method of Conway [17]. Briefly, after the ground sample (1 g) was added to 9 mL of double distilled (dd)-water and blended with a homogenizer (T-25 basic; IKA Labortechnik, Staufen, Germany) for 1 min, the mixture was filtered using a filter paper (Whatman #2; GE healthcare, UK). Filtered sample (1 mL) was injected into the outer chamber of a Conway cell, whereas boric acid solution (0.01 N, 1 mL) and indicator solution (0.066% methylene red and 0.066% bromocresol green, 50 μL) were injected into the inner chamber. Saturated K_2_CO_3_ solution (50%, 1 mL) was then injected into outer chamber. The chamber was sealed and incubated at 37°C for 120 min. A HCl solution (0.01 N) was titrated to samples until color turned to violet red. VBN values were derived by substituting titrated amount of HCl solution to the formula as followed:


$$\text{VBN value }(\text{mg}\%)=\frac{Titrated\;0.01N\;HCl\;solution\;(mL)\times 0.14\times dilutionfactor\times 100}{Sample\;weight}$$

### Microbiological analysis

Homogenized sausages (10 g) and sterilized dd-water (90 mL) were mixed to prepare microbial counts samples. Total plate count (TPC) agar for counting total bacteria and violet red bile agar for counting *Enterobacteriaceae* were prepared. A 0.1 mL of diluted sample was spread on agar in Petri dish and incubated at 37°C for 36 to 48 h. After incubating, the number of developed microbial colonies was counted. Microbial counts were presented as log colony-forming units per gram (log CFU/g).

### Statistical analysis

The whole experiments for this study were carried out in triplicate (n = 3). Preparation of pork sausages was performed three times in the same condition, and all analyses of parameter for this study were carried out per each manufacturing process. The IBM SPSS Statistics 23 (SPSS, Chicago, IL, USA) was used for calculating the mean and standard deviation. Data of all experiments except for CL were subjected to two-way analysis of variance (ANOVA) by two factors of 8 treatment groups (REF, CTL, TRT1, and TRT2 for both packaging methods) ×storage time (0, 2, 4, and 8 wks) as fixed effects. Three replicates were considered to be random effects (three times of sausages preparations and measurements of all parameters) for analyses. One-way ANOVA was performed for CL taking treatment as a fixed effect. If there were no interactions between the two factors in all experiments, data were pooled by treatments or storage time. If interaction was significant (p<0.05), data were separated by treatment within a storage time and by storage time within a treatment. Multiple comparisons (Post-hoc) were carried out by Duncan’s multiple range test at 5% of significance level.

## RESULTS AND DISCUSSION

### pH and color values

Table 2 shows pH and CIE color values of pork sausages added with different levels of salt, STE content, and rigor state of raw meat during 8 wks of storage with two different packaging methods. Since no interactions (treatment groups× storage time) in results of pH or color values were observed (p>0.05), data were pooled by treatment in a storage time and by storage time in a treatment. There were no differences in pH or color values among all treatments or packaging types (p>0.05), indicating that different levels of salt, STE, and rigor state of raw meat did not affect pH or color values of sausages with two packaging methods (p>0.05). From the 4th wk of storage time, pH values of sausages became lower than those of the initial storage period. These results showed that pH values of sausages might be affected by microbial growth during refrigerated storage. Therefore, pH values might decrease from the 4th wk of storage with increased microbial counts. In a previous study of Seo et al [18], pH values of pork sausages showed a decreasing trend during refrigerated storage of 5th wks. Deda et al [19] reported that reduction of pH in meat products was partially due to growth of lactic acid bacteria. Although we did not measure lactic acid bacteria in this study, increases of total bacterial counts might be due to increases of lactic acid bacteria known to reduce pH values of products and lead to spoil the products thereafter. Villamonte et al [20] reported that no differences were observed in lightness and redness of cooked pork meat batter between 1.5% and 3% salt levels. These results suggested that different salt levels might not influence color values of meat products.

### Cooking loss

**Figure 1:**
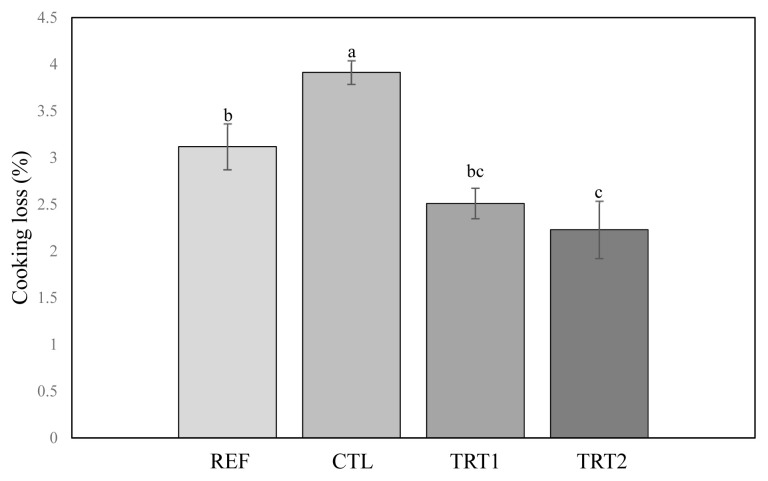
Cooking loss of pork sausages as affected by salt level, sea tangle extract content, and rigor state of raw meat. Treatments: REF, pork sausage (PS) added with 1.5% salt using post-rigor muscle; CTL, PS added with 0.8% salt using pre-rigor muscle; TRT1, PS added with 0.8% salt and 5% sea tangle extract (STE) using pre-rigor muscle; TRT2, PS added with 0.8% salt and 10% STE using pre-rigor muscle. ^a–c^ Means having the same superscripts are not different (p>0.05).

Results of CL (%) are shown in Figure 1. CL values of CTL were the highest among all treatments. Although the salt addition level of TRT2 (0.8%) was lower than that of REF (1.5%), CL values of REF were higher than those of TRT2 (p<0.05). Generally, the addition of higher salt into meat products can reduce the CL. However, the rigor state (pre-rigor vs post-rigor) and the addition of STE might affect CL more than added salt level (0.7%) in this study, resulting in the increase of CL value of REF (1.5% salt with post-rigor) than the TRT2 (0.8% salt level with pre-rigor and 10% STE). It is known that pre-rigor state of raw meat can decrease CL of cooked sausage due to the better functionality. Especially, in the 10% STE addition (TRT2), sausages manufactured with pre-rigor muscle and 0.8% salt were lower CL that those with post-rigor with 1.5% salt addition, as shown in Figure 1 (p<0.05). Roth et al [21] reported that sausages made with pre-rigor raw meat showed decreased CL due to increased water-holding capacity from the meat with higher pH values. Although CL values of TRT1 with 5% STE were similar to those of REF (p>0.05), those of TRT2 with 10% STE were lower than those of the REF (p<0.05). This result indicated that 10% STE could improve water-holding capacity of pork sausages during cooking and storage. Sea tangle contains alginate (a dietary fiber) and hydrocolloid as a functional molecular structure. It has been used in many foods to improve rheological properties, such as viscosity and gel formation ability [22]. Since it can cause reaction of electrostatic force with meat protein by calcium and gel, it might increase water-holding capacity [23,24]. Our results suggested that STE might contribute to decrease in CL of low-fat pork sausages and that the combination of STE with pre-rigor muscle could improve the water-holding capacity of reduced-salt meat products as compared to those made with 1.5% of salt addition level and post-rigor muscle.

### Texture profile analysis

Textural properties of sausage samples according to treatment and storage time are shown in Table 3. Since texture properties showed no interaction between the two factors, data were pooled by treatment or by storage time. Hardness values of all treatments didn’t differ by packaging methods (VAP vs MAP) (p>0.05). However, TRT2 had higher hardness values than CTL and REF with both VAP and MAP (p<0.05). Springiness values of all treatments were not different (p>0.05). Only REF with VAP had higher gumminess values than CTL (p<0.05), whereas TRT1 and TRT2 with MAP had higher gumminess values than the CTL (p<0.05). Chewiness values of TRT2 were higher than those of CTL (p<0.05). This trend was observed with both VAP and MAP. Kim et al [12] reported that the addition of 3% or 4% sea tangle powder into pork sausages increased gumminess values. This might be partially due to the effect of dietary fiber in sea tangle [25,26], which contains alginic acid, a water-soluble dietary fiber [27]. These results suggested that STE could improve textural properties of low-fat reduced salt pork sausages as compared to the control. However, no marked differences in texture properties were observed between two packaging methods (VAP vs MAP).

### Expressible moisture

Table 3 shows results of EM (%) values of pork sausages as affected by the rigor state, salt, and STE level during refrigerated storage. No differences in EM values of the same treatment with different packaging methods were observed (p>0.05). EM values of CTL were higher than those of other treatment groups (p<0.05). However, TRT1 and TRT2 with the same rigor state and salt levels had lower EM values than CTL (p<0.05). Despite lower additional levels of salt in TRT2, EM values of TRT2 with VAP were lower than those of REF (1.5% salt) (p<0.05). This result indicated that combination of pre-rigor muscle and STE could prevent the release of water during cooking, thus improving water-holding capacity of low-fat pork sausages. Choi et al [14] reported that the addition of sea tangle and sea mustard into frankfurter sausages improved their water-holding capacity, similar to results of this study. Seaweed such sea tangle and sea mustard contain dietary fiber including alginic acid and laminarain [28]. Thus, dietary fiber would be suitable for improving water-holding capacity of meat products by occupying the fiber with moisture [29,30].

### Purge loss

As shown in Table 4, PLs (%) of all treatments with MAP were lower than those with VAP (p<0.05). It means that MAP could decrease PLs of pork sausages in comparison with VAP. Cayuela et al [31] reported that MAP samples of pork loin had lower weight losses than VAP samples during storage (p<0.05). As a result, MAP could maintain the water binding ability and improve product quality during storage [32]. PLs of CTL with VAP were higher than those of REF in the same packaging condition (p<0.05). PLs of STE treated pork sausages were lower than STE untreated samples (p<0.05). PLs of TRT1 and TRT2 added with STE were similar to each other (p>0.05). However, they were lower than those of REF and CTL (p<0.05). These results indicated that TRT1 and TRT2 had higher water-holding capacity than those of REF and CTL, resulting in similar moisture loss during storage due to the addition of STE. According to Choi et al [13], dietary fibers of sea tangle (*Laminaria japonica*) such as alginate and laminarin improved water-holding capacities of reduced-fat pork patties. Robertson and Eastwood [33] reported that dietary fiber bound with water within the cell matrix. Dietary fiber and protein in meat system can affect quality characteristics such as water binding property of meat products [34]. In the present study, the addition of STE into low-fat sausages maintained the water holding ability without losing water during storage.

### Thiobarbituric acid reactive substances

As shown in Table 4, although TBARS (mg MDA/kg) values of all treatments with VAP were not different (p>0.05), those of TRT2 with MAP were lower than those of CTL in the same packaging condition (p<0.05). These results indicated that the addition of STE at a level of at least 10% of water extract into pork sausages could decrease TBARS values. Kim et al [35] reported that water extracts of sea tangle possessed antioxidant activity from vitamin E. Oh and Lim [36] reported that sea tangle powder decreased TBARS values of hamburger patties and suggested that sea tangle powder retarded lipid oxidation of pork patties. Park et al [37] reported that enzymatic hydrolysates of sea tangle increased activity of antioxidative enzymes such as catalase and glutathione, suggesting that sea tangle could contribute to the inhibition of lipid oxidation in meat products.

### Volatile basic nitrogen

Volatile basic nitrogen (mg/100 g) values of pork sausages as affected by rigor state, salt level, and STE level are presented in Table 4. Sausages with MAP showed lower VBN values than those with VAP (p<0.05). Regardless of packaging methods, VBN values of TRT1 and TRT2 were lower than those of CTL (p<0.05). Jeon and Choi [28] reported that VBN values of pork patties added with seaweed powder were lower than those of untreated control samples. They suggested that the addition of sea algae could extend shelf-life by improving antimicrobial and antioxidant activities of those pork patties. These results indicated that MAP could improve shelf-life by inhibiting protein degradation by retarding microbial growth in food during storage. Hur et al [38] suggested that VBN values of beef with MAP (CO_2_:N_2_ = 30:70) were lower than those packed in ziplock bags at 4th and 8th wks of cold storage. These results were similar to those of present study, indicating that MAP might inhibit protein degradation as compared to the VAP. In the present study, the addition of STE in combination with MAP might have potential to extend the shelf-life by retarding protein degradation and preventing moisture loss.

### Microbiological analysis

As shown in Table 4, TPC of TRT2 were lower than those of CTL with both VAP and MAP (p<0.05). REF with VAP showed higher TPC values than TRT2 (p<0.05). Although the reduction of salt decreased the antimicrobial effect of TRT2, decreases in TPC might be due to the addition of STE. Sea algae such as sea tangle and sea weed have been reported to have antimicrobial effect on *Bacillus subtilis* and *Escherichia coli* [39]. Thus, they might retard the growth of certain microorganisms. Lee et al [15] reported that emulsified sausages added with 3% sea tangle powder showed less total aerobic bacterial counts than the control under a sodium pyrophosphate-free condition. This indicated that the addition of sea tangle into sausages could exert antimicrobial activity since alginate contained in sea tangle could be an antimicrobial agent by inhibiting the growth of *Escherichia coli* [40] and *Staphylococcus aureus* [10]. These results were similar to the present study. Thus, reducing salt level at about 50% of original salt level in sausage products containing pre-rigor meat with STE could extend the shelf-life of meat products.

## CONCLUSION

The combination of pork pre-rigor muscle and STE could improve textural properties and water-holding capacity of reduced-salt (<1.0%) pork sausages. The addition of STE extended shelf-life of low-fat pork sausages by retarding protein and fat oxidation and inhibiting microbial growth. Therefore, low-fat pork sausages added with STE in combination with the use of pre-rigor raw meat could be successfully prepared as reduced-salt sausages. The additional level of salt could be reduced from 1.5% to 0.8%.

## Figures and Tables

**Table 1:** Formulation for manufacture of pork emulsified-sausages with different contents of oleoresin paprika solution

Ingredients (%)	Treatments^1)^			

	REF	CTL	TRT1	TRT2
Pork ham	60.0	60.0	60.0	60.0
Water	34.23	34.23	29.23	24.23
Non meat ingredients				
Sodium chloride	1.27	0.57	0.57	0.57
Sodium tripolyphosphate	0.40	0.40	0.40	0.40
Sodium erythorbate	0.05	0.05	0.05	0.05
Cure blend^2)^	0.25	0.25	0.25	0.25
Sugar	1.00	1.00	1.00	1.00
Spices	1.00	1.00	1.00	1.00
Fat replacer				
- Soy protein isolate	1.50	1.50	1.50	1.50
- Konjac and carrageenan	1.00	1.00	1.00	1.00
Sea tangle extract	0.00	0.00	5.00	10.0
Total	100.7	100.0	100.0	100.0

1) Treatments: REF, pork sausage (PS) added with 1.5% of salt using post-rigor muscle; CTL, PS added with 0.8% of salt using pre-rigor muscle; TRT1, PS added with 0.8% of salt and 5% of sea tangle extract using pre-rigor muscle; TRT2, PS added with 0.8% and 10% of sea tangle extract using pre-rigor muscle.

2) Cure blend consisted of 93.75% of salt and 6.25% of sodium nitrite.

**Table 2:** pH and color (CIE Lab*) values of pork sausages as affected by salt level, sea tangle extract content, and rigor state of raw meat

Treatments^1)^		pH	CIE L*	CIE a*	CIE b*
VAP	REF	5.98±0.22^a^	66.4±2.00^a^	9.42±1.27^a^	7.36±1.35^a^
	CTL	5.97±0.31^a^	68.1±1.35^a^	9.72±0.90^a^	7.77±0.91^a^
	TRT1	6.08±0.09^a^	67.8±1.39^a^	10.2±0.97^a^	8.34±1.06^a^
	TRT2	6.06±1.21^a^	67.4±0.68^a^	10.3±1.00^a^	8.46±0.78^a^
MAP	REF	5.99±0.20^a^	66.6±1.61^a^	9.40±1.37^a^	7.43±1.21^a^
	CTL	6.02±0.25^a^	67.8±1.29^a^	9.92±0.85^a^	7.99±1.23^a^
	TRT1	6.04±0.15^a^	67.7±1.04^a^	10.3±0.86^a^	8.59±1.26^a^
	TRT2	6.02±0.22^a^	67.3±0.34^a^	10.4±0.82^a^	8.62±0.90^a^
Storage time (wks)					
0		6.11±0.09^a^	67.7±1.47^a^	9.60±1.05^a^	8.00±1.12^a^
2		6.11±0.13^a^	67.0±1.42^a^	10.0±1.22^a^	7.99±1.26^a^
4		5.98±0.17^b^	67.3±1.20^a^	10.0±0.94^a^	8.17±1.32^a^
8		5.88±0.27^b^	67.5±1.40^a^	10.1±0.96^a^	8.13±1.01^a^

CIE, Commission Internationale de l’Eclairage; VAP, vacuum packaging; MAP, Modified atmosphere packaging.

1) Treatments: REF, pork sausage (PS) added with 1.5% of salt using post-rigor muscle; CTL, PS added with 0.8% of salt using pre-rigor muscle; TRT1, PS added with 0.8% of salt and 5% of sea tangle extract using pre-rigor muscle; TRT2, PS added with 0.8% and 10% of sea tangle extract using pre-rigor muscle.

a,b Means having the same superscripts in the same column are not different (p>0.05).

**Table 3:** Texture properties and expressible moisture values of pork sausages as affected by salt level, sea tangle extract content, and rigor state of raw meat

Treatments^1)^		Hardness (gf)	Springiness (mm)	Gumminess	Chewiness	Expressible moisture (%)
VAP	REF	4,028±676^bc^	5.05±1.04^a^	33.5±13.0^ab^	165±34.4^abc^	25.8±2.41^b^
	CTL	3,619±724^c^	4.96±0.60^a^	30.8±9.19^c^	147±39.7^c^	29.5±2.84^a^
	TRT1	4,637±1,396^ab^	5.37±1.03^a^	42.0±25.1^abc^	176±48.3^abc^	25.7±1.73^bc^
	TRT2	5,041±1,347^a^	5.26±1.06^a^	43.8±25.5^abc^	187±44.0^a^	23.6±1.80^c^
MAP	REF	4,088±698^bc^	4.78±0.85^a^	36.6±12.6^abc^	173±38.1^abc^	26.0±3.61^b^
	CTL	3,574±728^c^	5.02±0.83^a^	30.2±10.1^c^	151±39.6^bc^	30.3±2.00^a^
	TRT1	4,780±1,352^ab^	4.79±0.98^a^	46.2±25.6^ab^	185±54.7^ab^	25.2±1.88^bc^
	TRT2	5,047±1,625^a^	4.79±1.05^a^	49.7±26.6^a^	198±16.5^a^	24.0±2.31^bc^
Storage time (wks)						
0		5,437±156^a^	4.33±1.22^b^	59.7±27.1^a^	215±43.7^a^	26.9±2.79^a^
2		4,366±112^b^	4.81±0.86^b^	37.7±14.9^b^	163±43.5^b^	25.7±2.85^a^
4		3,706±622^c^	5.41±0.47^a^	26.9±5.58^c^	147±25.6^b^	26.8±3.49^a^
8		3,897±505^bc^	5.42±0.53^a^	32.1±8.05^bc^	166±35.3^b^	25.6±3.69^a^

VAP, vacuum packaging; MAP, modified atmosphere packaging.

1) Treatments: REF, pork sausage (PS) added with 1.5% of salt using post-rigor muscle; CTL, PS added with 0.8% of salt using pre-rigor muscle; TRT1, PS added with 0.8% of salt and 5% of sea tangle extract using pre-rigor muscle; TRT2, PS added with 0.8% and 10% of sea tangle extract using pre-rigor muscle.

a–c Means having the same superscripts in the same column are not different (p>0.05).

**Table 4:** Purge loss (%), thiobarbituric reactive substances (TBARS, mg MDA/kg), volatile basic nitrogen (VBN, mg/100 g), total plate counts (TPC, log CFU/g), and Enterobacteriaceae counts (violet red bile, log CFU/g) of pork sausages as affected by salt level, sea tangle extract content, and rigor state of raw meat

Treatments^1)^		Purge loss	TBARS	VBN	TPC	VRB
VAP	REF	3.86±1.77^b^	0.45±0.02^ab^	17.6±11.2^ab^	3.24±2.17^ab^	<2.00^a^
	CTL	4.98±1.86^a^	0.47±0.02^a^	19.4±12.2^a^	3.42±2.23^a^	<2.00^a^
	TRT1	3.43±0.89^c^	0.46±0.02^ab^	16.3±9.69^bc^	3.13±2.12^abc^	<2.00^a^
	TRT2	3.24±1.85^cd^	0.45±0.04^ab^	15.3±8.92^cd^	2.64±2.23^c^	<2.00^a^
MAP	REF	2.66±0.76^d^	0.45±0.03^ab^	14.9±8.45^cd^	3.20±2.07^ab^	<2.00^a^
	CTL	3.27±1.12^cd^	0.47±0.04^a^	16.3±9.52^bc^	3.45±2.18^a^	<2.00^a^
	TRT1	2.11±1.10^e^	0.45±0.03^ab^	14.1±8.04^d^	3.13±2.06^abc^	<2.00^a^
	TRT2	1.96±0.66^e^	0.44±0.03^b^	13.5±7.82^d^	2.88±2.02^bc^	<2.00^a^
Storage time (wks)						
0		-	0.44±0.02^b^	0.44±0.02^a^	<2.00^d^	<2.00^b^
2		2.32±0.79^a^	0.46±0.04^a^	0.46±0.04^a^	3.24±0.99^c^	<2.00^b^
4		2.85±1.02^a^	0.46±0.03^a^	0.46±0.03^a^	3.88±0.63^b^	<2.00^b^
8		4.40±1.81^b^	0.46±0.03^a^	0.46±0.03^a^	5.54±0.18^a^	3.90±0.23^a^

VAP, vacuum packaging; MAP, modified atmosphere packaging.

1) Treatments: REF, pork sausage (PS) added with 1.5% of salt using post-rigor muscle; CTL, PS added with 0.8% of salt using pre-rigor muscle; TRT1, PS added with 0.8% of salt and 5% of sea tangle extract using pre-rigor muscle; TRT2, PS added with 0.8% and 10% of sea tangle extract using pre-rigor muscle.

a–e Means having the same superscripts in the same column are not different (p>0.05).

## References

[1] Desmond E (2006). Reducing salt: A challenge for the meat industry. Meat Sci.

[2] Ruusunen M, Puolanne E (2005). Reducing sodium intake from meat products. Meat Sci.

[3] Turck D, Castenmiller J, de Henauw S (2019). Dietary reference values for sodium. EFSA J.

[4] World Health Organization (2012). Guideline: Sodium intake for adults and children.

[5] World Health Organization (2019). Healthy diet.

[6] Verma AK, Banerjee R (2012). Low-sodium meat products: retaining salty taste for sweet health. Crit Rev Food Sci Nutr.

[7] Motycka RR, Bechtel PJ (1983). Influence of pre-rigor processing, mechanical tenderization, tumbling method and processing time on the quality and yield of ham. J Food Sci.

[8] Puolanne EJ, Terrell RN (1983). Effects of rigor-state, levels of salt and sodium tripolyphosphate on physical, chemical and sensory properties of frankfurter-type sausages. J Food Sci.

[9] Kim HJ, Yang EJ (2015). Optimization of hot water extraction conditions of Wando sea tangle (*Laminaria japonica*) for development of natural salt enhancer. J Korean Soc Food Sci Nutr.

[10] Lee HS, Suh JH, Suh KH (2000). Preparation of antibacterial agent from seaweed extract and its antibacterial effect. Korean J Fish Aquat Sci.

[11] Hwang JK, Hong SI, Kim CT, Choi MJ, Kim YJ (1998). Quality changes of meat patties by the addition of sea mustard paste. J Korean Soc Food Sci Nutr.

[12] Kim HW, Choi JH, Choi YS (2010). Effects of sea tangle (*Lamina japonica*) powder on quality characteristics of breakfast sausages. Korean J Food Sci Anim Resour.

[13] Choi YS, Choi JH, Han DJ (2012). Effects of *Laminaria japonica* on the physico-chemical and sensory characteristics of reduced-fat pork patties. Meat Sci.

[14] Choi YS, Kum JS, Jeon KH (2015). Effects of edible seaweed on physicochemical and sensory characteristics of reduced-salt frankfurters. Korean J Food Sci Anim Resour.

[15] Lee H, Choe J, Yong HI, Lee HJ, Kim HJ, Jo C (2018). Combination of sea tangle powder and high-pressure treatment as an alternative to phosphate in emulsion-type sausage. J Food Process Preserv.

[16] Sinnhuber RO, Yu TC (1977). The 2-thiobarbituric acid reaction, an objective measure of the oxidative deterioration occurring in fats and oils. J Oleo Sci.

[17] Conway EJ, Conway EJ (1947). Determination of volatile amines. Microdiffusion analysis and volumetric error.

[18] Seo HW, Kang GH, Cho SH, van Ba H, Seong PN (2015). Quality properties of sausages made with replacement of pork with corn starch, chicken breast and surimi during refrigerated storage. Korean J Food Sci Anim Resour.

[19] Deda MS, Bloukas JG, Fista GA (2007). Effect of tomato paste and nitrite level on processing and quality characteristics of frankfurters. Meat Sci.

[20] Villamonte G, Simonin H, Duranton F, Chéret R, de Lamballerie M (2013). Functionality of pork meat proteins: Impact of sodium chloride and phosphates under high-pressure processing. Innov Food Sci Emerg Technol.

[21] Roth DM, Mckeith FK, Brewer MS (1997). Processing parameter effects on textural characteristics of reduced-fat pork sausage. J Food Qual.

[22] Hwang JK (1996). Physicochemical properties of dietary fibers. J Korean Soc Food Sci Nutr.

[23] Imeson AP, Ledward DA, Mitchell JR (1977). On the nature of the interaction between some anionic polysaccharides and proteins.

[24] Bernal VM, Smajda CH, Smith JL, Stanley DW (1987). Interactions in protein/polysaccharide/calcium gels. J Food Sci.

[25] Yılmaz I (2004). Effects of rye bran addition on fatty acid composition and quality characteristics of low-fat meatballs. Meat Sci.

[26] Choi YS, Jeong JY, Choi JH (2008). Effects of dietary fiber from rice bran on the quality characteristics of emulsion-type sausages. Korean J Food Sci Anim Resour.

[27] Jo HG, Kim MJ, Cheong SH (2019). Sea tangle (*Saccharina japonica*), an edible brown seaweed, improves serum lipid profiles and antioxidant status in rats fed high-fat and high-cholesterol diets. J Appl Phycol.

[28] Jeon MR, Choi SH (2012). Quality characteristics of pork patties added with seaweed powder. J Korean Food Sci Anim Resour.

[29] Cofrades S, Guerra MA, Carballo J, Fernández-Martín F, Colmenero FJ (2000). Plasma protein and soy fiber content effect on bologna sausage properties as influenced by fat level. J Food Sci.

[30] Kim HJ, Paik HD (2012). Functionality and application of dietary fiber in meat products. J Korean Food Sci Anim Resour.

[31] Cayuela JM, Gil MD, Bañón S, Garrido MD (2004). Effect of vacuum and modified atmosphere packaging on the quality of pork loin. Eur Food Res.

[32] Arvanitoyannis IS, Stratakos AC (2012). Application of modified atmosphere packaging and active/smart technologies to red meat and poultry: a review. Food Bioproc Technol.

[33] Robertson JA, Eastwood MA (1981). An examination of factors which may affect the water holding capacity of dietary fibre. Br J Nutr.

[34] Cofrades S, López-López I, Solas MT, Bravo L, Jiménez-Colmenero F (2008). Influence of different types and proportions of added edible seaweeds on characteristics of low-salt gel/emulsion meat systems. Meat Sci.

[35] Kim YS, Kang CO, Kim MH, Cha WS, Shin HJ (2011). Contents of water extract for *Laminaria japonica* and its antioxidant activity. Korean Soc Biotechnol Bioeng J.

[36] Oh HK, Lim HS (2001). Quality characteristics of the hamburger patties with sea tangle (*Laminaria japonica*) powder and/or cooked rice. Food Sci Anim Resour.

[37] Park PJ, Kim EK, Lee SJ (2009). Protective effects against H2O2-induced damage by enzymatic hydrolysates of an edible brown seaweed, sea tangle (*Laminaria japonica*). J Med Food.

[38] Hur SJ, Jin SK, Park JH, Jung SW, Lyu HJ (2013). Effect of modified atmosphere packaging and vacuum packaging on quality characteristics of low grade beef during cold storage. Asian-Australas J Anim Sci.

[39] Oh CK, Oh MC, Kim SH, Lim SB, Kim SH (1998). Antimutagenic and antimicrobial effect of ethanol extracts from sea-mustard and sea-tangle. Korean J Fish Aquat Sci.

[40] Cha DS, Choi JH, Chinnan MS, Park HJ (2002). Antimicrobial films based on Na-alginate and κ-carrageenan. LWT-Food Sci Technol.

